# SMART: Unique Splitting-While-Merging Framework for Gene Clustering

**DOI:** 10.1371/journal.pone.0094141

**Published:** 2014-04-08

**Authors:** Rui Fa, David J. Roberts, Asoke K. Nandi

**Affiliations:** 1 Department of Electronic and Computer Engineering, Brunel University, Uxbridge, Middlesex, United Kingdom; 2 National Health Service Blood and Transplant, Oxford, United Kingdom; 3 The University of Oxford, John Radcliffe Hospital, Oxford, United Kingdom; 4 Department of Mathematical Information Technology, University of Jyväskylä, Jyväskylä, Finland; Universitat Rovira i Virgili, Spain

## Abstract

Successful clustering algorithms are highly dependent on parameter settings. The clustering performance degrades significantly unless parameters are properly set, and yet, it is difficult to set these parameters *a priori*. To address this issue, in this paper, we propose a unique splitting-while-merging clustering framework, named “splitting merging awareness tactics” (SMART), which does not require any *a priori* knowledge of either the number of clusters or even the possible range of this number. Unlike existing self-splitting algorithms, which over-cluster the dataset to a large number of clusters and then merge some similar clusters, our framework has the ability to split and merge clusters automatically during the process and produces the the most reliable clustering results, by intrinsically integrating many clustering techniques and tasks. The SMART framework is implemented with two distinct clustering paradigms in two algorithms: competitive learning and finite mixture model. Nevertheless, within the proposed SMART framework, many other algorithms can be derived for different clustering paradigms. The minimum message length algorithm is integrated into the framework as the clustering selection criterion. The usefulness of the SMART framework and its algorithms is tested in demonstration datasets and simulated gene expression datasets. Moreover, two real microarray gene expression datasets are studied using this approach. Based on the performance of many metrics, all numerical results show that SMART is superior to compared existing self-splitting algorithms and traditional algorithms. Three main properties of the proposed SMART framework are summarized as: (1) needing no parameters dependent on the respective dataset or *a priori* knowledge about the datasets, (2) extendible to many different applications, (3) offering superior performance compared with counterpart algorithms.

## Introduction

Clustering methods have been widely used in many fields, including biology, physics, computer science, communications, artificial intelligence, image processing, and medical research, requiring analysis of large quantities of data to explore the relationships between individual objects within the respective datasets [1]–[13]. However, clustering is one of the most difficult and challenging problems in the realm of machine learning due to the lack of universal and rigorous mathematical definition. The definition of clustering often depends on the specific systems or problems, e.g. in computer vision, where it is defined as image segmentation [1], [2], or in complex network analysis, where it is known as graph clustering or community detection [14]–[16].

After some pioneering works by Eisen et al. [6], Golub et al. [7], and Tamayo et al. [8], clustering was extensively employed in gene expression analysis where microarray and real time sequencing have allowed rapid measurement of genome-wide transcription[3], [5], [17]–[25]. There are many families of clustering algorithms used in the gene expression analysis, including partitional clustering, hierarchical clustering, model-based clustering, self-organizing clustering [3], [23]. Results of most of successful clustering algorithms strongly depend on the determined number of clusters, e.g. k-means, model-based clustering, and hierarchical clustering (when the clustering memberships need to be determined). However, in many cases, *a priori* knowledge of the actual number of clusters is not available. Thus, the number of clusters has to be estimated beforehand. The problem of determining the best number of clusters needs to be addressed in another branch of research in clustering analysis, known as clustering validation [26]–[28]. Among various clustering validation criteria, clustering validity indices, also known as relative criteria, have been employed to quantitatively evaluate the goodness of a clustering result and estimate the best number of clusters. There are two main classes of validity indices: a) model-based or information theoretic validation, e.g. minimum description length (MDL) [29], minimum message length (MML) [30], [31], Bayesian information criterion (BIC) [32], Akaike's information criterion (AIC) [33], and the normalized entropy criterion (NEC) [34]; b) geometric-based validation, which considers the ratio of within-group distance to between-group distance (or its reciprocal), such as Calinski-Harabasz (CH) index [35], *Dunn's* index (DI) [36], Davies-Bouldin (DB) index [37], *I* index [38], Silhouette index (SI) [39], the geometrical index (GI) [40], the validity index 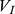 [41] and the parametric validity index (PVI) [42], [43].

Once an appropriate clustering validity index is selected, the general practice for determining the best number of clusters has few steps: a set of clustering results are firstly obtained by a clustering algorithm with fixed number of clusters within a predetermined range [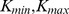]; then, these clustering results are evaluated by the chosen validity index; finally, depending on the chosen validity index, maximum or minimum index value indicates the best number of clusters (in some cases if the index value has an increase or decrease trend against the number of cluster, the significant knee point indicates the best number of clusters). However, this solution requires an extensive search for the number of clusters and is tedious work for large number of clusters

Moreover, the initialization of clustering is also a major issue. For some algorithms with the deterministic initialization, e.g. hierarchical clustering and clustering with kauffman approach initialization (KA) [44], the optimal solution is not always guaranteed. For some algorithms sensitive to initialization, such as k-means with random initialization, expectation-maximization (EM) [17], and self-organization map (SOM) [45], they may get stuck at local minimum. Addressing this problem requires running the algorithm repeatedly with the same dataset using several different initializations. This makes such clustering algorithms more computationally unfavourable. Thus, better options would be integrative frameworks or strategies which provide an automatic and consistent clustering, so users do not have to worry about setting those data-specific parameters.

Earliest attempts of automated clustering without employing any 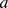 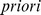 knowledge of number of clusters were growing cell structure [46] and growing neural gas [47]. Although these algorithms are useful to visualize high dimensional data, they are not suitable for clustering because they over-fit the data. A self-splitting competitive learning (SSCL) algorithm was proposed to achieve the automated clustering [48]. In SSCL, a competitive learning paradigm, so called *one-prototype-take-one-cluster* (OPTOC), was developed for self-splitting by employing an asymptotic property vector (APV) to guide the learning of a prototype; meanwhile a split validity criterion was embedded in SSCL to assess whether each cluster would contain more than one prototype: if it was the case, then cluster would be split into two. However, there are two vital issues to prevent its practical uses: 1) the prototypes are easily trapped into global centroid, especially the first few ones [48], and 2) the parameters for stopping both OPTOC learning and splitting are crucial to the algorithm but they are difficult to estimate reliably [49]. Yet, the SSCL has an attractive advantage in that it does not require *a priori* knowledge about the number of clusters in the input dataset.

Another strategy for automated clustering has been proposed using a similar method [49]–[52]. In these approaches, the input data was over-clustered to a large number of partitions, say 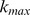, then these partitions were merged to fewer clusters, which were closer to the natural clusters. This strategy is called splitting-then-merging (STM). In terms of clustering techniques, the algorithm by Figueiredo and Jain [50] was based on unsupervised learning of finite mixture models (ULFMM), the self-splitting-merging competitive learning (SSMCL) by Wu and colleagues in [49] was based on OPTOC competitive learning paradigm, and a variational Bayesian Gaussian mixtures (VBGM) framework has been explored [51], [52]. Another critical difference between these algorithms is that the criteria for selecting final clustering are different. In ULFMM, along with the merging process from 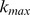 to 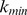, a model order selection criterion, which was minimum message length (MML) in their case, was used; in SSMCL, as a merging criterion was defined according to the measurement of distortion between two clusters, merging process would not stop until no cluster met the merging criterion; in VBGM, after the convergence of the optimization algorithm, the estimated number of clusters tends to be the number of non-empty clusters. There are two critical issues in the STM framework: one is that the maximum number of clusters 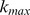 has to be determined *a priori*, however such an upper limit is subjective and sometimes only an inexact estimate is available; another issue is that as one of bottom-up algorithms, the STM framework cannot produce a very accurate clustering result in some circumstances, since it makes clustering decisions based on local patterns without initially taking into account the global distribution. Recently, Mavridis and colleagues proposed a parameter-free clustering (PFClust) algorithm, which is able to determine the number of clusters automatically [53]. PFClust clusters the dataset in two steps: first step is to estimate expectation and variance of intra-cluster similarity by randomisation; second step is to cluster the dataset based on the threshold calculated in randomisation. However, to select a suitable threshold, PFClust needs a good approximation to the distribution of mean intra-cluster similarities, and it requires a large number of randomisation which is time-consuming.

Here, we propose a new splitting-merging clustering framework, named “splitting-merging awareness tactics” (SMART) to overcome these problems. The proposed framework is different from aforementioned over-cluster-then-merge strategy and employs a novel splitting-while-merging (SWM) strategy. The proposed system integrates such crucial clustering techniques as cluster splitting methods, cluster similarity measurement, and clustering selection, within a framework to mimic human perception doing the sorting and grouping, which was inspired by the work of Zhang and Liu [48]. The framework starts with one cluster and accomplishes many clustering tasks to split and merge clusters. While splitting, a merging process is also taking place to merge the clusters which meet the merging criterion. In this process, SMART has the ability to split and merge clusters automatically in iterations. Once the stop criterion is met, the splitting process terminates and then a clustering selection method is employed to choose the best clustering from several generated ones. Moreover, the SMART framework is not restricted to a specific clustering technique. In this paper, we implement SMART in two algorithms using two distinct clustering paradigms: SMART I employs OTPOC competitive learning as the splitting algorithm and the calculation of cohesion between two clusters [54] as the merging criterion; and SMART II employs modified component-wise expectation maximization of mixtures (CEM^2^) [50], which was originally proposed in [55], to fulfil splitting and merging. For both algorithms, once the splitting-merging process terminates, a model order selection algorithm plays a critical role in selecting the best clustering among the generated clusterings during the splitting procedure. Two benchmark demonstration datasets are used to illustrate each step in the SMART flow. The main purpose of this paper is to develop the SMART framework and its algorithms for microarray gene expression datasets. Thus, two simulated gene expression datasets and two real microarray gene expression datasets are studied using SMART. By comparing the performance of several metrics, namely adjusted Rand index (ARI) [61], [62], correct selection rate (CSR) of number of clusters, the estimated number of clusters (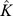), normalized mutual information (NMI), Jaccard index (JI), Silhouette index (SI), Calinski-Harabasz (CH) index, and minimum message length (MML), the numerical results show that our proposed method is superior. Most importantly, SMART does not require any parameters dependent on the respective dataset or *a priori* knowledge about the datasets.

The main sections of this paper are organised in the following sequence. The next section describes the philosophy of the proposed framework. We then provide the results of many examples, including two demonstration examples, two simulated datasets and two real gene expression datasets, to support the proposed framework. Subsequently, the clustering techniques employed in the SMART framework are detailed in Methods section. Finally, we conclude with a discussion of applications for future research.

## Results

### SMART Framework

First of all, we must emphasize that SMART is a framework rather than a simple clustering algorithm, within which a number of clustering techniques are organically integrated. Thus, conceptually, SMART does not fall into any categories classified in [2], [4]. In this section, we focus on the overview of the whole framework, and describe implementation solutions and specific clustering techniques in the following sections.

**Figure 1:**
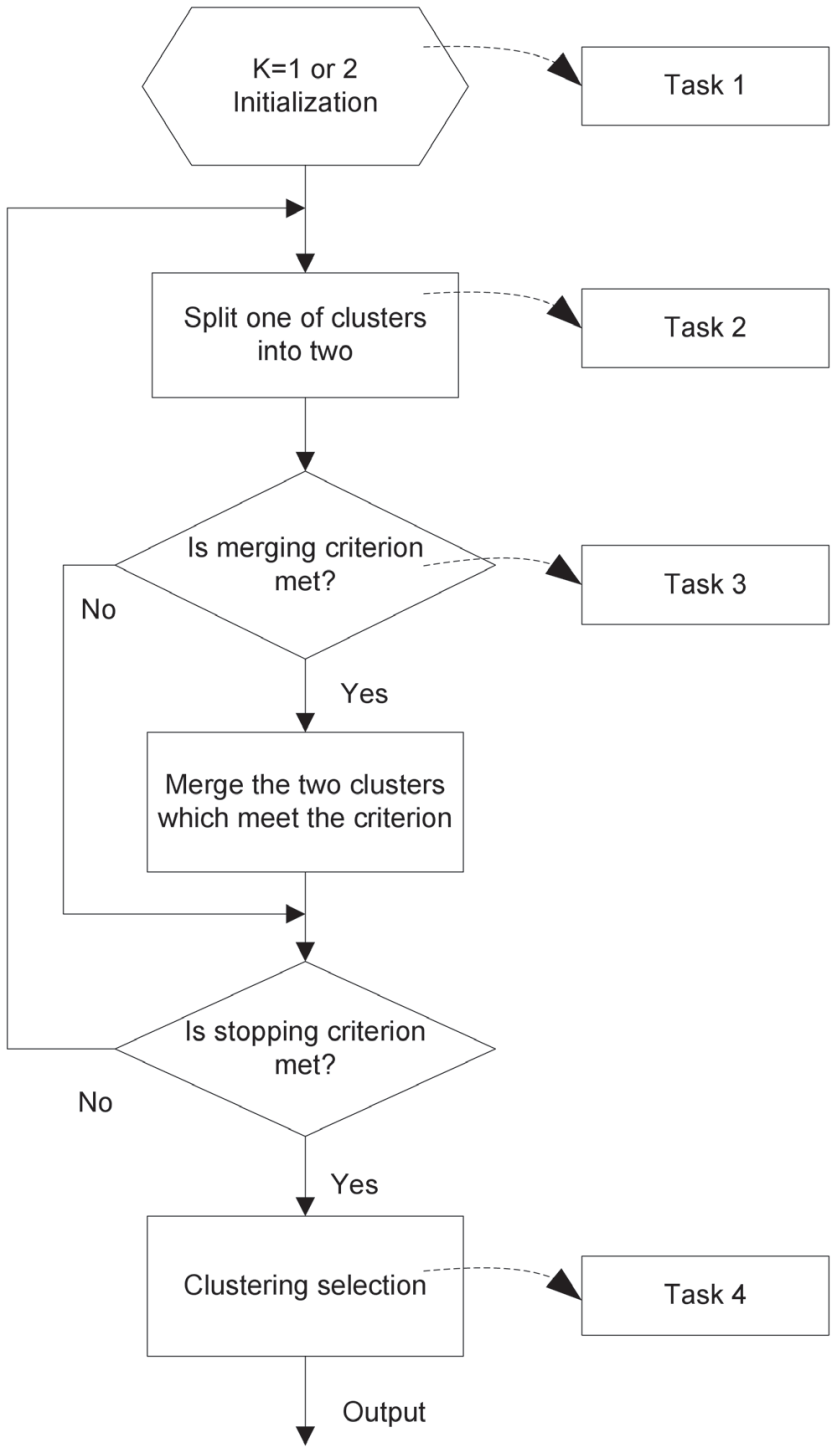
The flow chart of the SMART framework. SMART is initialized in Task 1; SMART splits one of clusters into two in Task 2; the new clustering is censored by a merging criterion in Task 3; SMART goes through the SWM process iteratively and generates many candidate clusterings; finally, the optimal clustering is selected by clustering selection criterion in Task 4.

Suppose that we are going to partition the dataset 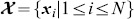, where 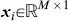 denotes the 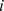-th object, 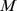 is the dimension, and 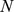 is the number of objects. The flowchart of the framework is illustrated in Fig. 1.

The whole clustering procedure is divided into four tasks. SMART starts with one cluster (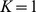, where 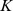 is the number of clusters), and the cluster needs to be initialized, which is Task 1. Subsequently, the data goes through a SWM process, where splitting and merging are automatically conducted in iterations. In the splitting step of each iteration, which is labelled Task 2, SMART splits one of the clusters into two. After a splitting step, the new clustering is censored by a merging criterion, which is associated with Task 3. If the condition for merging is satisfied, then one merges the two clusters, otherwise the merging step is skipped. SMART then goes through a termination-check, where a stopping criterion is applied. If the condition for termination is not satisfied, SMART goes to the next iteration and continues to split, otherwise, SMART finishes the splitting-merging process. The last step is the clustering selection (Task 4).

Note that these tasks in the SMART flow can be completed using many clustering techniques in the literature, e.g., Task 1 can be done by any initialization technique either deterministic or random; Tasks 2 and 3 may be achieved by any splitting algorithm and merging criterion respectively or they may be combined into one algorithm; and Task 4 can be accomplished by any of either model order selection algorithms or validity indices. Different techniques will make the implementation slightly different but the flow does not change. Moreover, different clustering algorithms bring different features into the framework and so SMART can be customized for different applications. In the following Methods section, we will develop two SMART algorithms using different splitting and merging algorithms, i.e., OPTOC competitive learning and finite mixture model learning, which are called SMART I and SMART II, respectively, and they have similar configurations. In particular, both use MML [30], [31] as clustering selection algorithm and use the same termination criterion in the SWM process, namely the maximum number of merges, 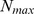. The logic behind the termination criterion is that normally merging will not start until optimal clustering is reached. Once 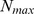 is reached, the splitting and merging will terminate automatically. We summarise the categorization of existing self-splitting-merging algorithms and our two SMART algorithms in Table 1. All existing self-splitting-merging algorithms employ the STM strategy with different clustering paradigms; instead our SMART algorithms employ the SWM strategy. For the purposes of direct comparisons with the existing STM algorithms, we propose two specific SMART algorithms. Nevertheless, it should be noted that, within the proposed SMART framework, many other algorithms can be derived for different clustering paradigms.

**Table 1:** Categorisation of two existing splitting-then-merging (STM) algorithms and our two splitting-while-merging (SWM) SMART algorithms.

	STM (requiring 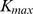 )	SWM
Competitive Learning	SSMCL	SMART I
Finite Model Mixtures (Gaussian)	ULFMM, VBGM	SMART II

### Experiment Set-up

In this paper, we use two demonstration datasets, which are bivariate mixture models: the first one is quadrature phase-shift keying (QPSK) data with signal-to-noise ratio (SNR) equal to 15 dB and the second one is a 3-component bivariate mixture [50]. Since we have more interest in the microarray gene expression data analysis, we employ two microarray gene expression data modelling methods to simulate or synthesize gene expression data. One simulates the state-based gene expression data [19] and another one simulates the periodic behaviour of yeast cell cycle[17], [59]. The advantages of using simulated data are that the ground truth is known and we have the freedom to manipulate the noise level of the data by tuning a few parameters. Additionally, two real microarray gene expression datasets are studied using SMART. The performance comparisons are carried out between the SMART algorithms and both SSMCL, ULFMM, VBGM, DBSCAN [60], MCLUST [17] and PFClust [53] in all experiments. Moreover, two state-of-the-art mixture model clustering, namely the mixture of factor analysers (MFA) [22] and the mixture of common factor analysers (MCFA) [21] are compared. Since these algorithms require a time-consuming exhaustive search over both a range of number of clusters (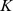) and a range of number of factors (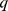), with a number of initial starts, we only compare them in real datasets. We list the software in which all clustering algorithms were implemented in Table 2. In our study, many metrics are investigated: ARI, CSR of number of clusters, the estimated number of clusters 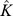, NMI, JI, SI, CH and MML, where both the mean and the standard deviation are presented for ARI, 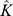, NMI, JI, SI, CH and MML. Note that for all metrics except 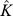 and MML, the maximal values are the measures of the best clustering results. CSR is the ratio of the times of the number of clusters being correctly selected, to the total number of experiments. In the following experiments, the parameters for SMART I and II are set as: 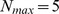 for both SMART I and II; 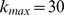 for SSMCL, ULFMM and VBGM. For MFA and MCFA, the parameters are set as: 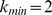, 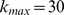, the number of factors 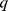 from 1 to 10, using 50 initial starts. For PFClust, we set the number of randomisation to be 10000. For MCLUST, we employ MML as clustering validation to estimate the number of clusters because it does not estimate the number of clusters automatically. For all demonstration datasets and simulated datasets, we feed them into clustering algorithms as they were generated without normalisation. Thus, the inputs for all algorithms are treated equally. Although we standardise each profile of gene to be zero mean and unit variance for real datasets, it is still the case that the inputs for all algorithms are treated equally.

**Table 2:** The list of Software with which all clustering methods in this paper are implemented.

Methods	Software	Reference
MFA	MATLAB	[22]
MCFA	MATLAB	[21]
SSMCL	MATLAB	[49]
ULFMM	MATLAB (Downloaded)	[50]
VBGM	MATLAB (Downloaded)	[52]
SMART I	MATLAB	-
SMART II	MATLAB	-
DBSCAN	R (FPC Package)	[60]
MCLUST	R (Mclust Package)	[17]
PFClust	Java (downloaded)	[53]

### Demonstration Examples

**Figure 2:**
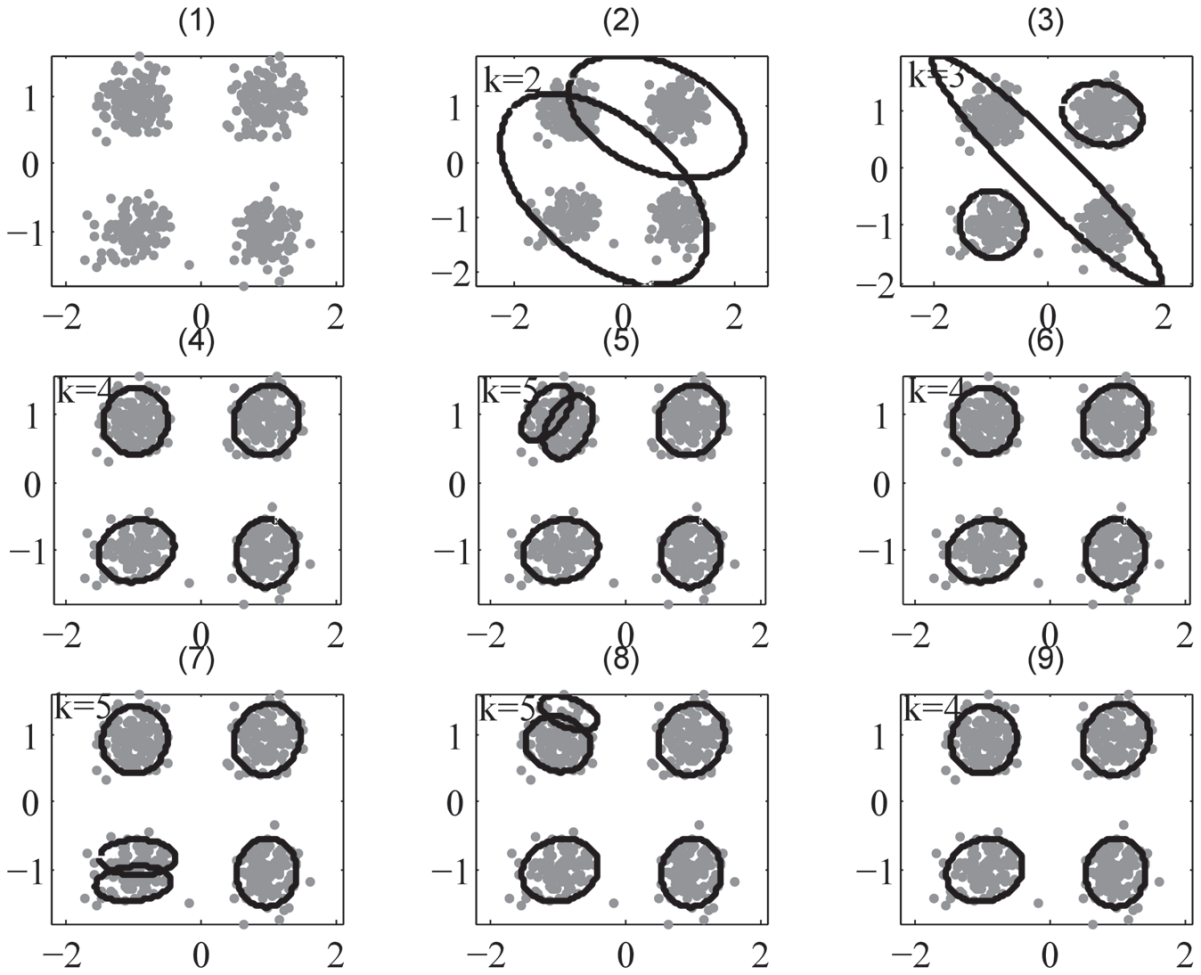
The demonstration of SMART I using QPSK dataset in D1 example. Sub-figures(1) – (8) demonstrate that the procedure of SMART I (SWM process). It starts with 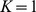 (sub-figure(1)), splits into 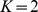. 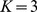, 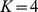 and 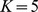 shown sub-figures(2) – (5) respectively, and then merges some clusters while splitting as shown in sub-figures(6) – (8). The sub-figure(9) is the final clustering result. Parameter settings: 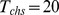 and 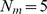.

**Figure 3:**
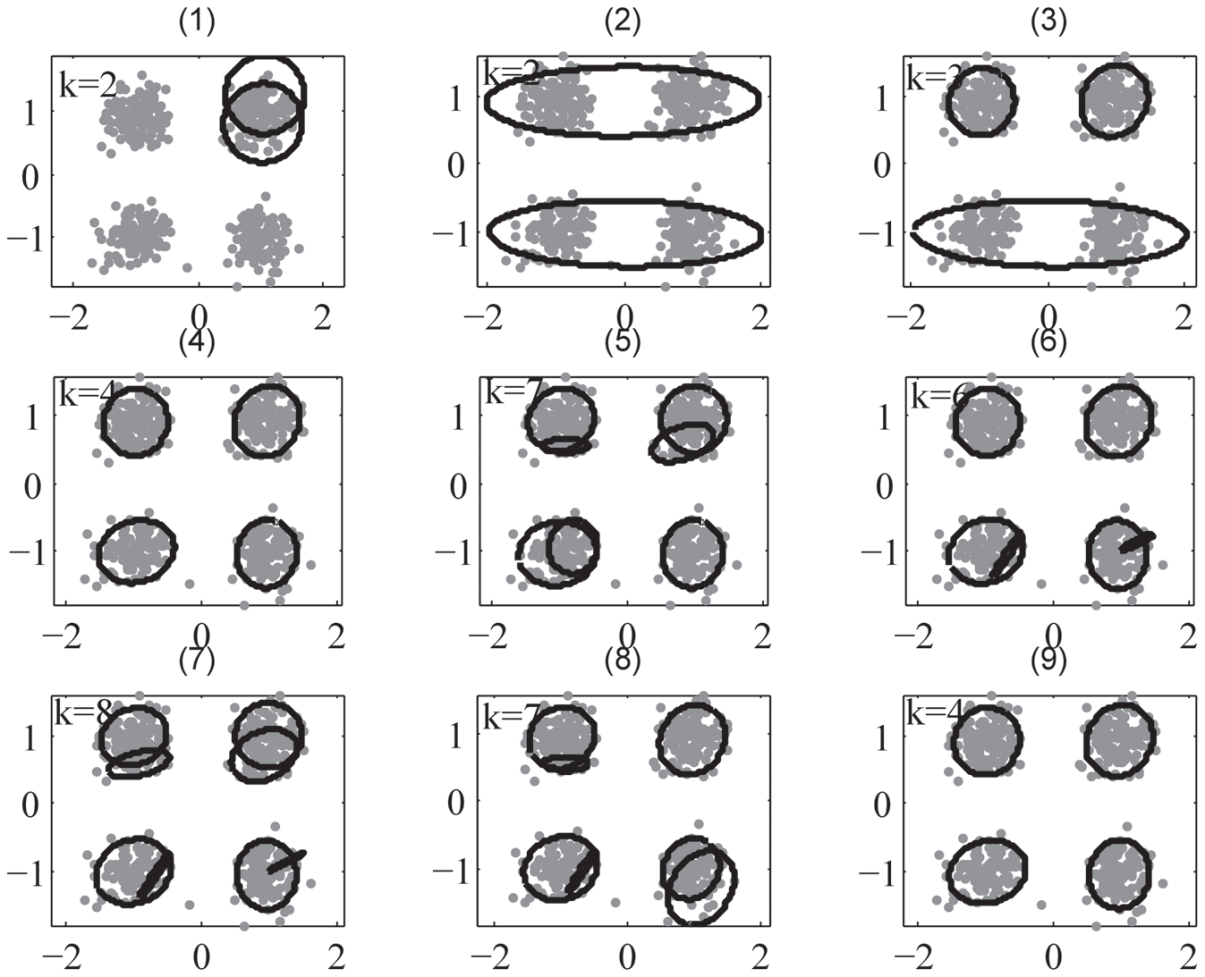
The demonstration of SMART II using QPSK dataset in D1 example. Sub-figures (1) – (8) demonstrate the procedure of SMART II. It starts with 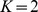 (sub-figure(1)), splits into 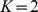. 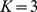, 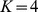 and 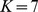 shown sub-figures(2) – (5) respectively, and then merges some clusters while splitting as shown in sub-figures(6) – (8). Sub-figure(9) is the final clustering result. Parameter setting: 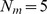.

**Figure 4:**
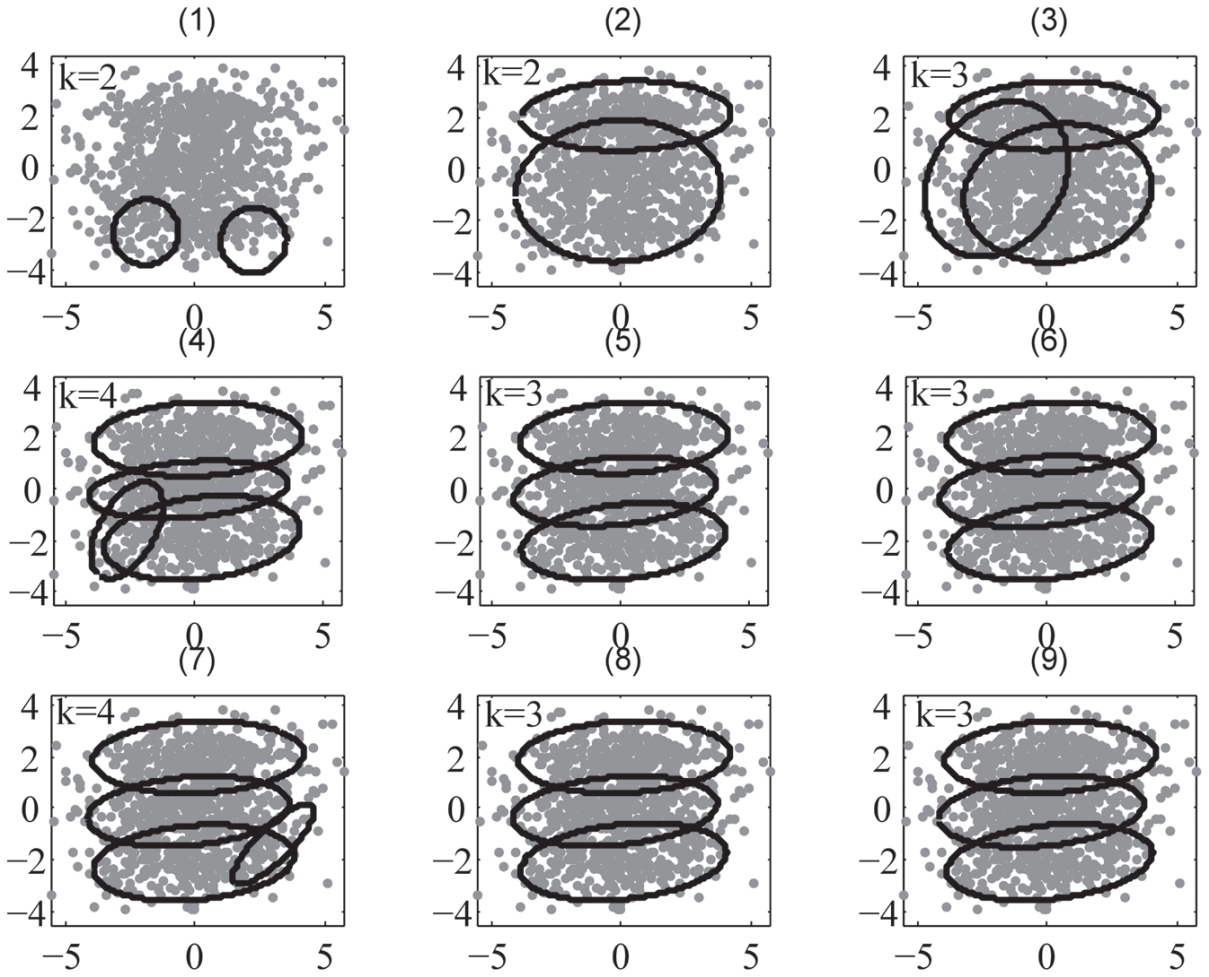
The demonstration of SMART II using Gaussian mixture dataset in D2 example. Sub-figures (1) – (8) demonstrate the procedure of SMART II. SMART II starts from 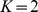 as shown in sub-figures(1) and (2), splits the dataset to 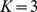 and 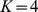 shown in sub-figures(3) and (4); the merging commences while splitting continues as shown in sub-figures(5) – (8). Sub-figure(9) is the final clustering result. Parameter setting: 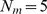.

**Figure 5:**
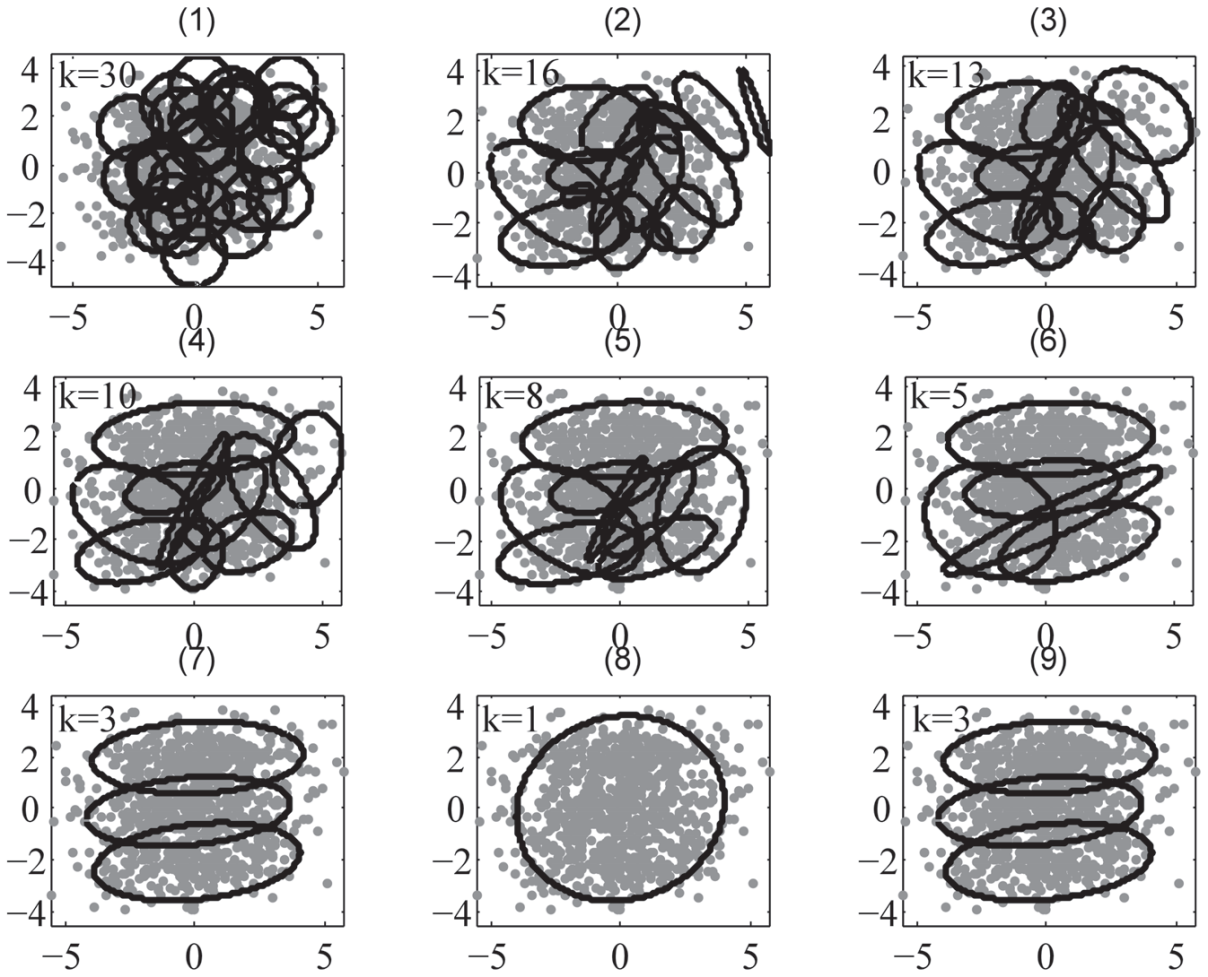
The demonstration of ULFMM using Gaussian mixture dataset in D2 example. Sub-figures (1) – (8) demonstrate the procedure of ULFMM. ULFMM starts from 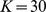 as shown sub-figure(1); ULFMM then merges clusters gradually to 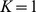 as shown in sub-figures(2) – (8) respectively. Sub-figure(9) is the final clustering result. Parameter setting: 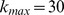.

In the first place, we employ a benchmark test dataset – 512-samples QPSK data with SNR level of 15 dB, which is labelled D1 dataset. This dataset can also be viewed as a 4-component Gaussian mixture. This example may clearly demonstrate how SMART I and II work, as shown in Figs. 2 and 3, respectively. In both Figs. 2 and 3, subfigures from (1) to (8) illustrate the proposed SWM process in the SMART framework, and subfigure (9) shows the final clustering result. The results show that the first merge of SMART I is after 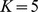 shown in Fig. 2-(5) and the first merge of SMART II is after 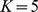 shown in Fig. 3-(5). Subsequently, the merge counter measures the times of merges until the SWM process terminates. To compare SMART with the state-of-the-art clustering algorithms, namely SSMCL, ULFMM, DBSCAN, MCLUST, PFClust and VBGM, using the same dataset, we repeat the clustering experiments 1000 times for each algorithm. The numerical results for D1 are shown in Table 3. SMART II, DBSCAN, MCLUST, PFClust and VBGM produce perfect results in all metrics, which means that there is no mis-clustered members at all in their results in the whole experiment. For other algorithms, the metrics are not always consistent. SSMCL has the poorest performance compared with other algorithms according to all metrics except that it has lower MML value than SMART I. SMART I provides higher values of CSR, SI, and CH, and has more closer mean and smaller standard deviation of 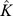 than ULFMM, but ULFMM has better performance in ARI, NMI, JI, and MML. The reason for this observation may be that SSMCL occasionally put some objects into wrong clusters but the number of clusters is correct, while ULFMM sometime wrongly splits an actual cluster into two but the objects are mostly in the correct clusters.

**Table 3:** Performance comparison of many metrics, including ARI, CSR, 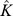, NMI, JI, SI, CH, MML, for all algorithms in D1.

Algorithms	ARI	CSR	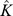	NMI	JI	SI	CH	MML
SSMCL	0.993 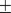 0.03	70.2%	4.3 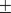 0.5	0.995 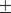 0.03	0.993 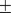 0.05	0.902 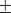 0.04	1.2E3 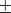 147	779.9 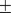 24.4
ULFMM	0.998 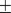 0.01	93.2%	4.1 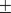 0.5	0.998 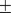 0.01	0.998 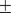 0.01	0.898 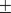 0.10	1.26E3 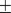 112	779.8 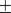 2.0
VBGM	**1** 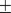 **0**	**100%**	**4.0** 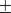 **0**	**1** 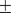 **0**	**1** 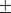 **0**	**0.923** 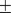 **0**	**1.29E3** 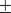 **0**	**779.6** 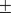 **0**
DBSCAN	**1** 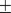 **0**	**100%**	**4.0** 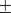 **0**	**1** 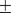 **0**	**1** 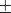 **0**	**0.923** 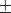 **0**	**1.29E3** 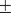 **0**	**779.6** 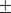 **0**
MCLUST	**1** 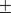 **0**	**100%**	**4.0** 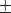 **0**	**1** 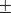 **0**	**1** 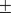 **0**	**0.923** 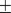 **0**	**1.29E3** 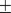 **0**	**779.6** 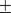 **0**
PFClust	**1** 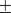 **0**	**100%**	**4.0** 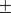 **0**	**1** 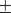 **0**	**1** 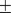 **0**	**0.923** 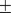 **0**	**1.29E3** 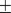 **0**	**779.6** 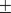 **0**
SMART I	0.994 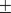 0.02	98.6%	4.0 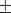 0.2	0.996 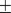 0.03	0.995 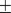 0.02	0.918 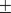 0.05	1.28E3 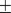 125	782.2 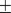 38.3
SMART II	**1** 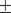 **0**	**100%**	**4.0** 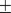 **0**	**1** 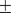 **0**	**1** 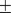 **0**	**0.923** 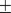 **0**	**1.29E3** 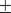 **0**	**779.6** 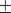 **0**

The second demonstration example D2 is a 3-component bivariate Gaussian mixture dataset used in [50], whose mixture probabilities are 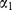  =  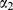  =  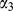  = 1/3, with mean vectors at 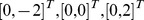, and equal covariance matrices 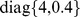. The covariance matrices are 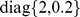 in [50], but we double them in our study as we try to discern the best algorithm by enlarging the differences among their performances. The numerical results for D2 dataset are shown in Table 4. SSMCL and SMART I fail in this experiment. The reason is that the competitive learning is a spherical or hyper-spherical algorithm so it is not suitable for the clustering of elliptical or hyper-elliptical datasets. Although SMART I has higher CH and SI values than both SMART II and ULFMM, other metrics all reveal that SMART I performs poorly. SMART II has 100% CSR in the experiment and other performance in all metrics are best except CH and SI. The explanation of this observation is that CH and SI use Euclidean distance, which is a hyper-spherical metric. Thus CH and SI are not reliable in this case. It is also worth noting that VBGM has much poorer performance than SMART II in this case, in particular, only 72.4% CSR. These results reflect that SMART II is much better than ULFMM and VBGM where there is considerable noise. DBSCAN fails in this experiment can does not cluster at all (resulting all-zero partition); MCLUST and PFClust perform poorly in this dataset. The clustering procedures of SMART II and ULFMM are shown in Fig. 4 and 5, respectively. These two demonstration examples show how the mechanism of SMART is working. To some extent, they also show that the SMART framework is more effective and more practical than ULFMM, because it is not necessary for SMART to set 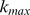.

**Table 4:** Performance comparison of many metrics, including ARI, CSR, 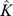, NMI, JI, SI, CH, MML, for all algorithms in D2.

Algorithms	ARI	CSR	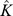	NMI	JI	SI	CH	MML
SSMCL	0 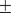 0	0.0%	1 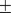 0	0 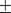 0	0 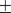 0	/	/	/
ULFMM	0.64 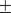 0.03	88.1%	3.2 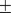 0.7	0.61 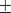 0.02	0.61 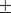 0.02	0.27 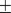 0.11	138.3 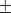 21.4	3.68E3 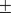 5.6
VBGM	0.60 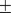 0.05	72.4%	3.77 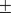 1.3	0.49 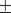 0.04	0.44 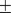 0.05	0.23 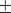 0.05	134.3 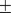 16.6	3.70E3 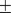 17.4
DBSCAN	-	-	-	-	-	-	-	-
MCLUST	0.25 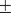 0.0	0.0%	12 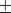 0.0	0.44 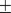 0.0	0.23 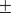 0.0	0.40 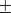 0.0	304.2 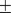 0.0	3.78E3 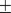 0.0
PFClus	0.16 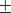 0.0	0.0%	2.0 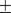 0.0	0.15 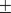 0.0	0.33 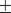 0.0	0.33 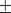 0.0	197.3 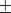 0.0	3.80E3 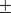 0.0
SMART I	0.19 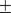 0.15	14.6%	3.3 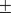 1.4	0.23 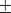 0.17	0.33 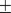 0.06	**0.43** 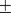 **0.06**	**412.4** 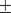 **70.3**	3.72E3 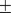 263.5
SMART II	**0.70** 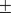 **0.01**	**100%**	**3.0** 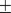 **0**	**0.62** 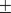 **0.01**	**0.63** 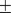 **0.01**	0.30 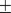 0.003	145.8 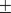 0.2	**3.68E3** 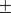 **0.2**

### Simulated Gene Expression Datasets

The first experiment (S1) is a stochastic model which simulates the state-based gene expression data [19]. There are 11 clusters 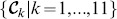 of genes with 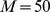 samples in the simulated data. The cluster size 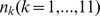 satisfy Poisson distribution 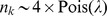. The expression values are simulated as a hierarchical log-normal model in each cluster. For 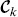, firstly, a vector of cluster template for the cluster is created with four periods of expression of size 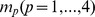. The sizes of 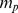 are from a uniform distribution such that 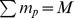 and 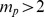. The initial template in four periods is simulated from 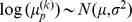. Secondly, sample variability (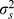) is introduced and the gene sample template 
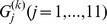
 is generated from 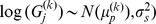, where 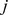 is such that 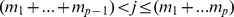. Then for each gene vector 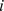 in sample 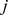, the gene variability is added and expression values are generated as 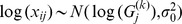. Lastly, once gene data is simulated, a random noise from normal distribution (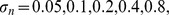 and 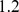) is added. The parameters used in this model are set as: 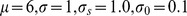, and 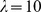. We generate 100 datasets for each 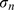.

**Figure 6:**
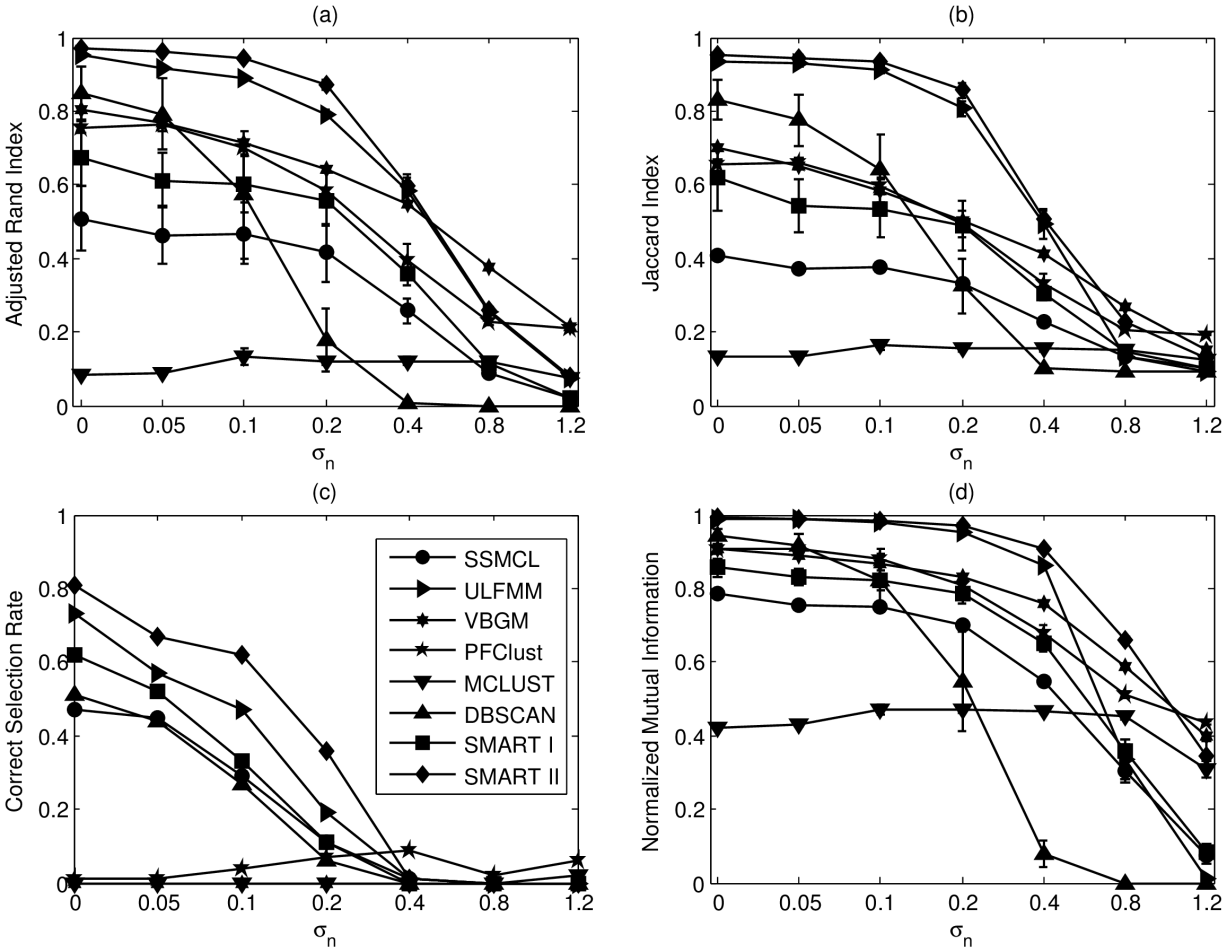
The errorbar charts of (a) ARI, (b) JI, (c) CSR, and (d) NMI for all compared algorithms in S1 datasets. The values of all four metrics are in the range of [0,1], where 1 is the optimal value and 0 is the worst one. The vertical axis in each sub-figure represents individual index and the horizontal axis is the standard deviation 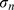 of the additive noise. SMART I and II are labelled with square and diamond markers respectively. SSMCL is labelled with circle marker, ULFMM is labelled with right-arrowed triangle marker, VGBM is labelled with by hexagon marker, PFClust is labelled with pentagon marker, MCLUST is labelled with down-arrowed triangle marker, and DBSCAN is labelled with up-arrowed triangle marker. For SMART I, 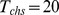; for SMART I and II, 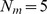. For ULFMM, SSMCL, and VBGM, 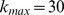.

**Figure 7:**
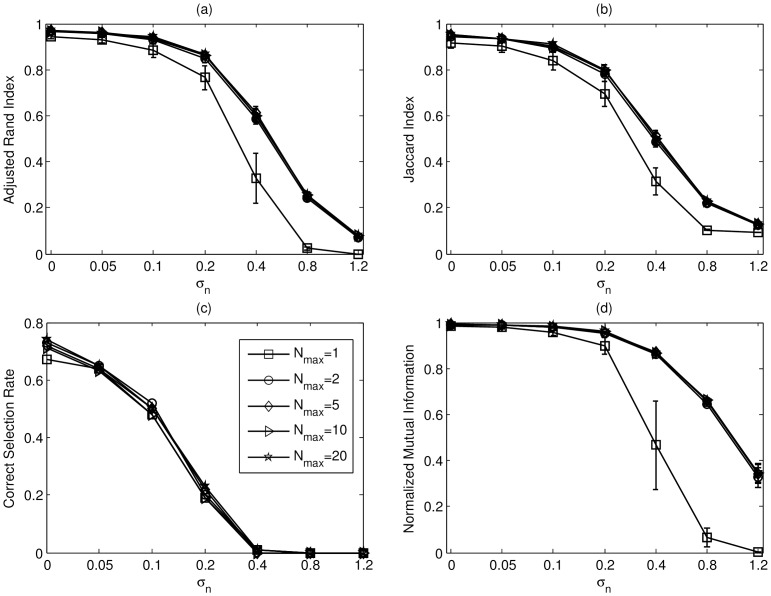
The errorbar charts of (a) ARI, (b) JI, (c) CSR, and (d) NMI for SMART II with different 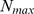 values in S1 datasets. The vertical axis in each sub-figure represents individual index and the horizontal axis is the standard deviation 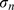 of the additive noise. The line with square markers denotes 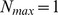;The line with circle markers denotes 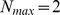;The line with diamond markers denotes 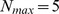;The line with triangle markers denotes 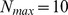;The line with pentagon markers denotes 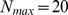.

The errorbar charts of ARI, JI, CSR, and NMI are shown in Figs. 6 (a) – (d) respectively. Generally speaking, in S1, it is found that the FMM clustering works better than the competitive learning and that the SMART framework has better performance than over-cluster-then-merge strategy. The proposed SMART II algorithm has superior performance when the noise level is low or moderate. It has above 60% CSR and ARI, JI, and NMI values close to 1 when the noise variance 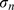 is equal or smaller than 0.1. In all noise levels where 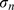 is below 0.4, SMART II always provides the superior performance among the compared algorithms and no algorithm works well when 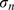 is greater than 0.4. We also investigate the impact of the parameter 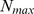 on the performance of SMART in S1 datasets, and the results are shown in Fig. 7. It is worth noting that the performance of SMART is stable when 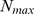 is greater than or equal to two; in other words, the performance of SMART is not sensitive to the value of 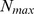.

**Figure 8:**
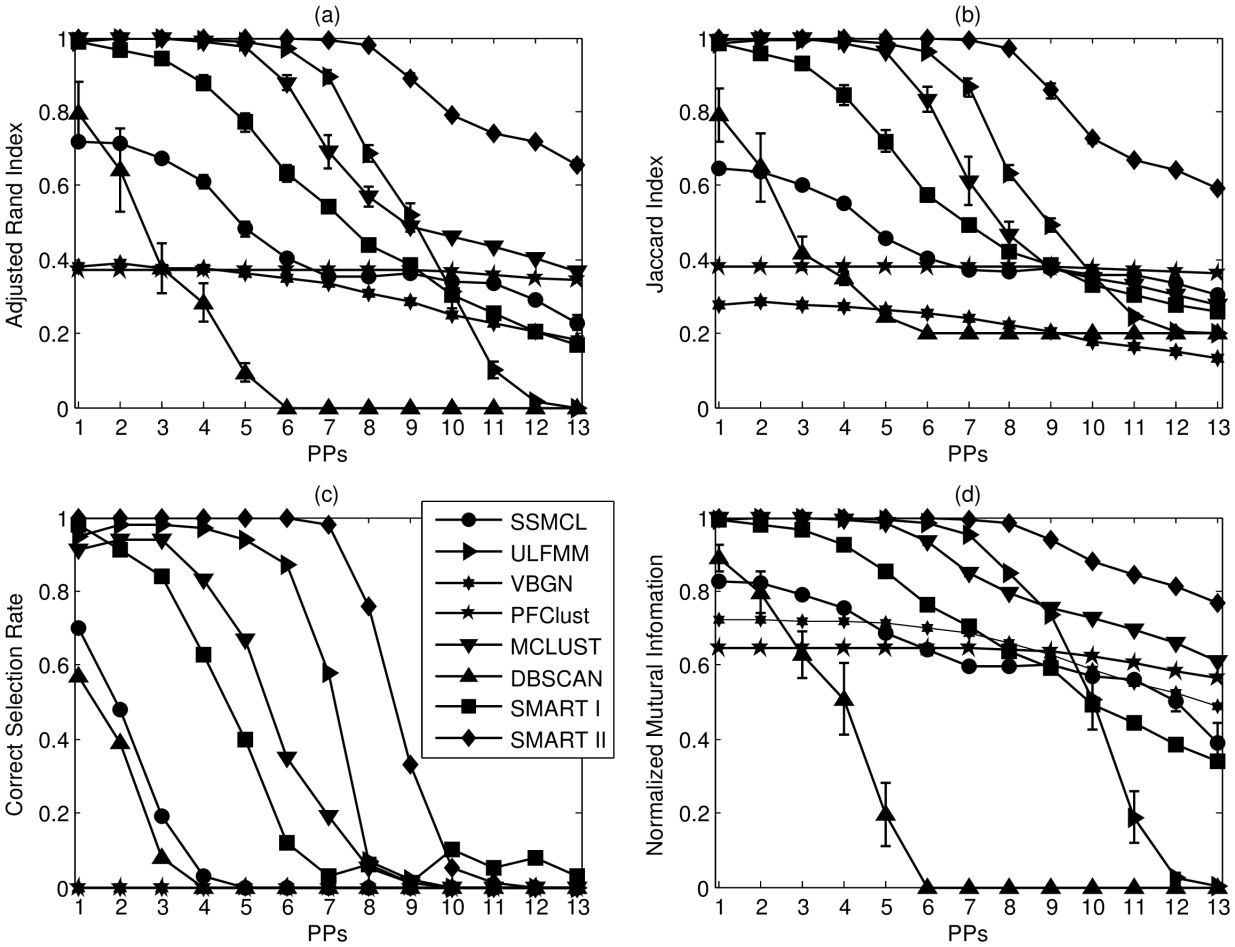
The errorbar charts of (a) ARI, (b) JI, (c) CSR, and (d) NMI for all compared algorithms in S2 datasets. The vertical axis in each sub-figure represents individual index and the horizontal axis is parameter pairs from PP1 to PP13, representing 13 noise levels from low to high. For SMART I, 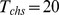; for SMART I and II, 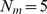. For ULFMM, SSMCL, and VBGM, 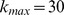.

**Figure 9:**
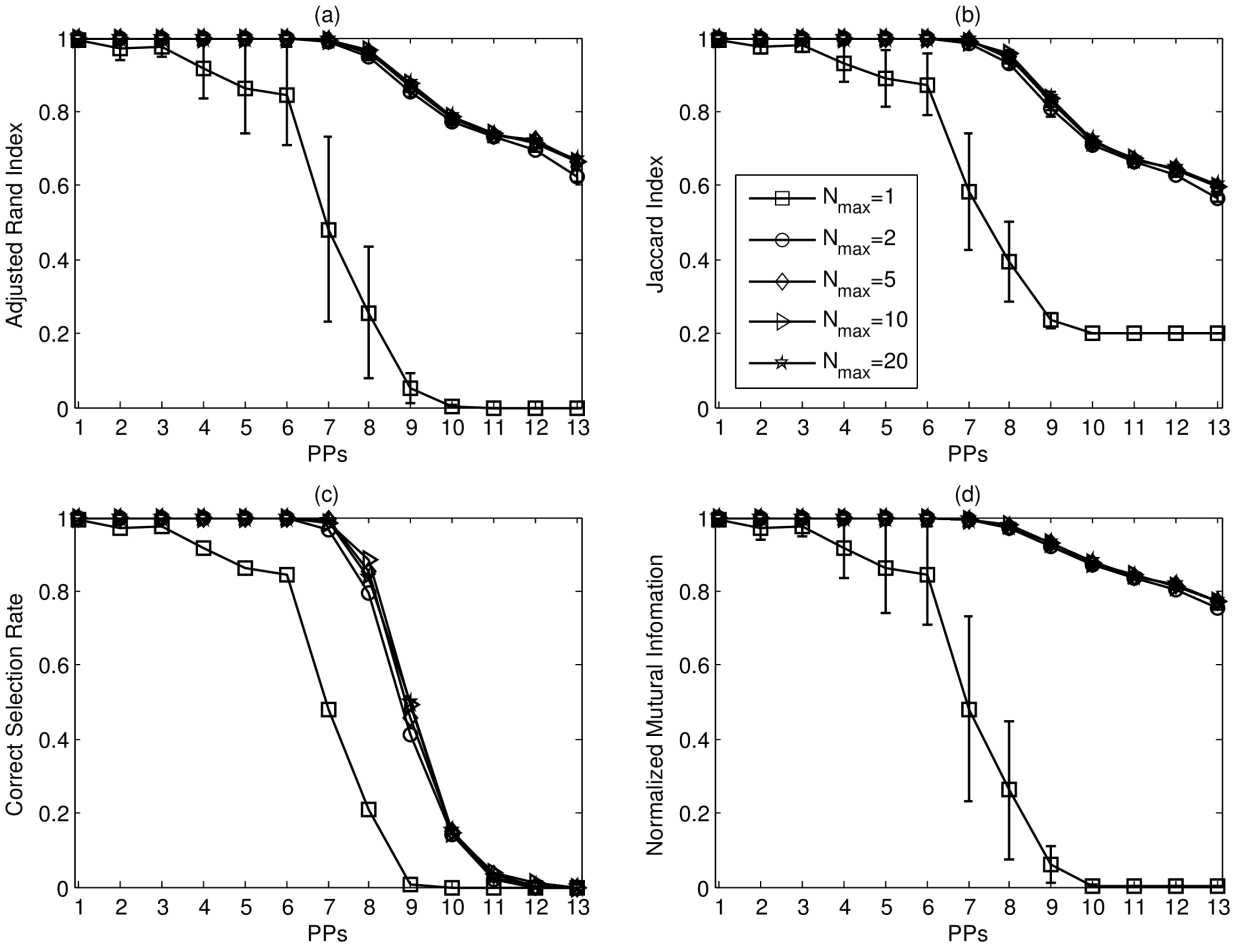
The errorbar charts of (a) ARI, (b) JI, (c) CSR, and (d) NMI for SMART II with different 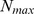 values in S2 datasets. The vertical axis in each sub-figure represents individual index and the horizontal axis is parameter pairs from PP1 to PP13, representing 13 noise levels from low to high.

In the second simulated dataset experiment (S2), we employ the method in [59] to generate a number of synthetic gene expression datasets with 500 synthetic genes in each dataset and 24 samples for each gene. These 500 genes belong to 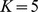 clusters and each cluster has 100 members. The model of cyclic gene expression is given by 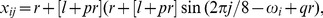
(1)where 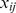 is the expression value of the 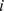-th gene at the 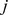-th time point, each instant of 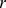 is an independent random number from the standard normal distribution 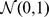, the parameter 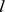 controls the magnitude of the sinusoid and it is fixed to three here. The parameter 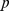 controls the random component added to the magnitude and the parameter 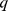 controls the random component added to the phase. The parameter 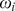 is the phase shift of the 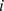-th gene and will determine which cluster the gene 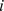 will be in. Since the noise in this model is not additive, we have to couple 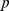 and 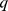 to be a pair, and raise both their values to change the noise power. By increasing values of 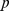 and 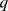 will increase the noise power increases. The paired parameters are listed as 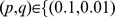, 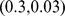, 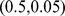, 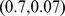, 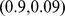, 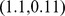, 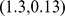, 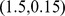, 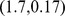, 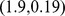, 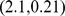, 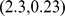, 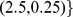. Thus, there are 13 parameter pairs (PPs) from PP1 to PP13 representing 13 noise levels from low to high. For each pair of parameters, we generate 100 datasets, and subsequently, we get 100 clustering results from each clustering algorithm. Figs. 8 (a) – (d) respectively show the errorbar charts of ARI, JI, CSR, and NMI achieved by each method in the S2 experiment. The results lead to the similar conclusion obtained in S1 experiment, which is namely FMM clustering works better than competitive learning and the SMART framework has better performance than over-cluster-then-merge strategy. The most impressive observation is that the proposed SMART II algorithm shows all ARI, JI and NMI values equal to one and 100% CSR until the 7-th PP, which is 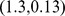, while no other method has 100% CSR performance and no other method has comparable performance in the whole experiment. We carry out the same investigation of impact of the parameter 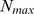 as in the S1 datasets. The results are shown in Fig. 9, which also indicate that the performance of SMART is not sensitive to the value of 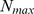.

### Real Microarray Gene Expression Datasets

Although the simulated experiments may have the advantage that they show different performance in different conditions for each method, they suffer the crucial drawback that they are not real. So we have tested our SMART using real datasets.

The first real dataset (R1) is a subset of the leukemia dataset [7], which consists of 38 bone marrow samples obtained from acute leukemia patients at time of diagnosis. There are 999 genes in the dataset [63]. The biological truth is that the samples include 3 groups: 11 acute myeloid leukemia (AML) samples, 8 T-lineage acute lymphoblastic leukemia (ALL) samples and 19 B-lineage ALL samples [7], [63], [64]. We repeat the clustering experiments 1000 times for each method. We also compare two state-of-the-art mixture model clustering algorithms, namely MFA and MCFA, with our proposed SMART algorithms. Since these algorithms require a time-consuming exhaustive search over a range of 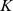 and a range of 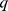 with a number of initial starts, we run them only once for each 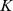 and each 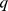 with 50 initial starts, where 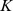 ranges from 2 to 30 and 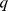 ranges from 1 to 10. The results are shown in Table 5. SSMCL and VBGM totally fail in this experiment, where SSMCL always converges to one cluster and VBGM always terminates at 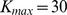. Impressively, SMART I has significantly better performance than ULFMM and has nearly 30% greater CSR and better performance in other metrics. In terms of mean and standard deviation of 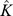, SMART I has a mean closer to the true value and significantly smaller standard deviation than ULFMM. Both MFA and MCFA have their lowest MML values with three clusters, but compared with two SMART algorithms, they show poorer performance in all metrics. SMART II has the superior performance and always provides 100% CSR and best performance in all other metrics. Particularly, SMART II also has very small variations in these metrics, that is, it provides consistent results even though it is randomly initialized. In this experiment, DBSCAN, MCLUST, and PFClust perform poorly and do not provide the correct estimates of the true number of clusters. Furthermore, their other validation metrics are worse than the SMART II algorithm. We have also examined the impact of variable values of 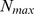 on the performance. We choose three values for the testing, 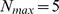, 10, and 20. The results are shown in Table 6. We can read from the Table that in all performance metrics, there is no significant difference among the results from different 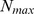 values. Thus, It confirms again that the SMART algorithms are not sensitive to the parameter 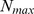 in this test.

**Table 5:** Performance comparison of many metrics, including CSR, 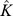, MML, CH, SI for all algorithms in Leukemia dataset.

Algorithms	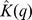	CSR	MML	CH	SI
MFA	3 (7)	/	4.23E4	6.42	0.35
MCFA	3 (4)	/	4.22E4	6.48	0.35
SSMCL	1 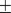 0	0.0%	/	/	/
ULFMM	3.23 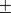 0.54	69.4%	3.91E4 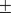 2.07E2	5.96 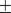 0.89	0.32 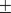 0.06
VBGM	30 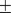 0	0.0%	4.02E4 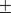 2.27E3	0.78 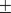 0.02	0.048 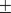 0.013
DBSCAN	1 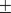 0	0.0%	/	/	/
MCLUST	2 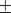 0	0.0%	4.27E4 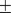 0.0	6.73 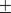 0.0	0.36 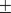 0.0
PFClust	4 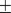 0	0.0%	4.31E4 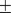 2.91	3.73 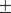 4.3E-3	0.21 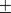 2.51E-4
SMART I	2.99 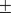 0.13	99.0%	3.89E4 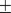 1.62E2	6.49 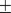 0.3	0.36 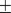 0.02
SMART II	**3 ** 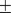 ** 0**	**100%**	**2.9E4** 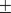 **8.37E-3**	**6.75** 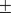 **5.64E-5**	**0.36** 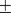 **5.81E-8**

**Table 6:** Performance comparison of SMART I and II with variable values of 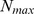.

		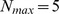	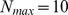	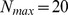
**SMART I**	MML	3.89E4 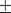 1.62E2	4.00E4 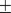 1.52E2	4.00E4 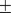 2.01E2
	CSR	99%	98.4%	98.4%
	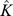	2.99 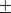 0.13	2.98 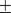 0.15	2.98 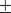 0.15
	CH	6.49 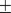 0.3	6.49 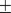 0.13	6.49 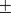 0.12
	SI	0.36 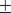 2E-2	0.35 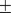 9.8E-3	0.35 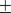 1.2E-2
**SMART II**	MML	2.9E4 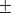 8.37E-4	3.27E 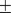 1.97E-3	3.26 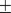 1.76E-3
	CSR	100%	100%	100%
	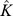	3 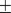 0	3 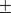 0	3 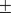 0
	CH	6.75 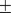 5.64E-5	6.55 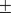 8.37E-5	6.55 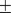 6.11E-5
	SI	0.36 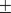 5.81E-8	0.36 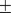 5.83E-8	0.36 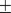 5.83E-8

**Figure 10:**
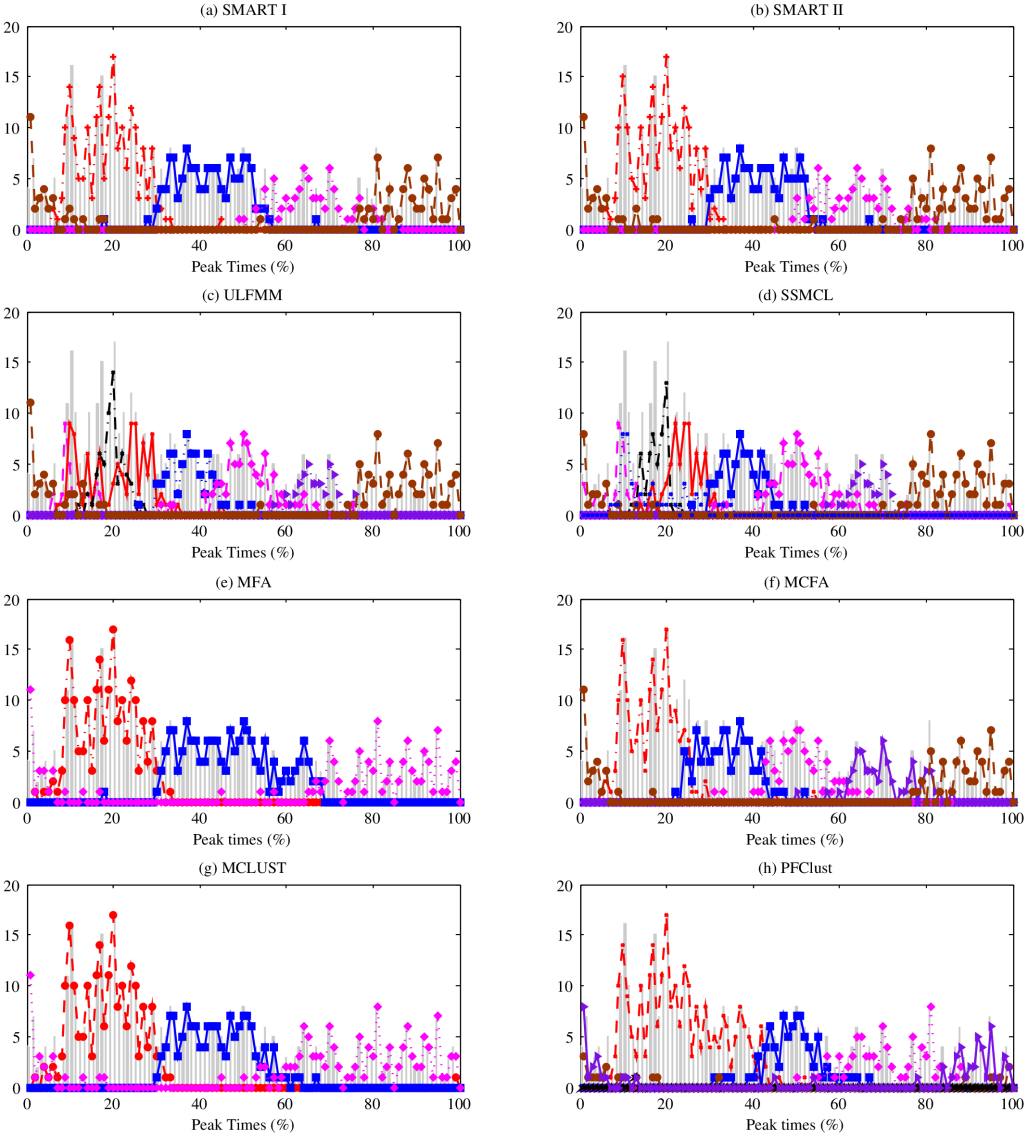
Histogram of the peak times of genes in each cluster for each algorithm in Yeast cell cycle 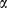-38 dataset. (a) SMART I, 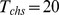 and 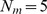, 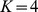 (b) SMART II, 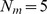, 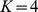, (c) ULFMM,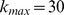, 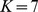, (d) SSMCL,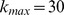, 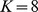, (e) MFA, 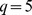, 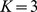, (f) MCFA, 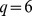, 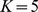, (g) MCLUST, 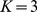, (h) PFClust. Sub-figures (a) and (b) show that four clusters represent reasonably good clustering since there are only few small overlap regions between clusters. Sub-figures (c) and (d) indicate that many clusters crowd and overlap in the region of 5% to 30%, especially in Sub-figure (c), a clustering representing peaking at 20% superposes on another cluster, which spans over 10% to 30%. These overlapped clusters have to be one cluster. Sub-figures (e) and (f) show that MFA and MCFA also give reasonably good clustering results judged by eye, however poorer than SMART II in the numerical metrics. Sub-figures (g) and (h) show the distribution of the peak times of genes based on the clustering results of MCLUST and PFClust, respectively.

Another real dataset (R2) is yeast cell cycle 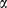-38 dataset provided in Pramila *et al.* [65]. It consists of 500 genes with highest periodicity scores and each gene has 25 time samples. Additionally, their peaking times as percentages of the cell cycle have also been provided by Pramila et al. [65]. It is widely accepted that there are four phases in the cell cycle, namely, G1, S, G2 and M phases [66], [67]. But there is no explicit knowledge about how many clusters should be in this dataset, so we cannot calculate CSR in this case. We obtain four clusters by using both SMART I and II, seven clusters by using ULFMM, eight clusters using SSMCL, three clusters using MFA with five factors, and five clusters using MCFA with six factors, as shown in Table 7. SMART II has the superior performance as in other experiments. We note that VBGM fails again in this experiment as it requires a dimension reduction of the data before clustering. We do not perform a reduction in data dimensions to obtain a fair comparison. To discern the effectiveness of the clusterings, we plot the histogram of the peak times of genes in each cluster for each algorithm, as depicted in Fig. 10, where the grey bar plot is the histogram of the 500 genes in the dataset. Fig. 10 (a) and (b) show that four clusters represent reasonably good clustering since there are only few small overlap regions between clusters. Fig. 10 (c) and (d) indicate that many clusters crowd and overlap in the region of 5% to 30%, especially in Fig. 10 (c), a clustering representing peaking at 20% superposes on another cluster, which spans over 10% to 30%. These overlapped clusters have to be one cluster. Fig. 10 (e) and (f) show that MFA and MCFA also give reasonably good clustering results judged by eye, however poorer than SMART II in the numerical metrics. Fig. 10 (g) and (h) show the distribution of peak times of genes based on the clustering results of MCLUST and PFClust, respectively. MCLUST has a very similar performance to MFA. The partition provided by PFClust has a cluster (labelled by brown circle) overlapping with other clusters. The numerical metrics consistently indicate that PFClust performs poorly in the R2 dataset. Since DBSCAN and VBGM do not provide a reasonable result, we do not depict it in Fig. 10. The results reveal that the SMART algorithms, especially, SMART II, provide a better representation than other algorithms.

**Table 7:** Performance comparison of many metrics, including 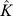, MML, CH, SI for all algorithms in yeast cell cycle dataset.

Algorithms	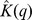	MML	CH	SI
MFA	3 (5)	1.36E4	6.68	0.37
MCFA	5 (6)	1.30E4	6.49	0.37
SSMCL	8	2.11E4	3.82	0.14
ULFMM	7	1.23E4	6.03	0.38
VBGM	20	3.97E4	1.98	0.17
DBSCAN	1	/	/	/
MCLUST	3	1.394	6.46	0.38
PFClust	6	1.24E4	3.94	0.32
SMART I	4	1.26E4	6.27	0.37
SMART II	4	**1.16E4**	**6.86**	**0.39**

We also compare the running time of the clustering algorithms for two real datasets in Table 8, where the algorithms implemented with MATLAB are listed in the upper section and the algorithms implemented with other platforms are in the lower section. For the sake of a fair comparison, we consider the running time of single run as the time consumed to find both best number of clusters and best partition, rather that the time only for clustering with one given number of clusters. The computer on which we conducted the experiments is equipped with Intel Core i7-3770 CPU 3.40 GHz and 8 GB RAM. According to the Table, SMART II consumed the least running time in both datasets. SMART I is faster that its counterpart algorithm SSMCL, but slower than ULFMM and VBGM. MFA and MCFA are time-consuming because they have to exhaustively search over both a range of number of clusters (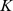) and a range of number of factors (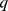), with a number of initial starts. The algorithms using other platforms, namely DBSCAN, MCLUST, and PFClust, also take longer time than the SMART algorithms to finish the same pieces of work.

**Table 8:** Comparison of running time (seconds) of the algorithms implemented in MATLAB (upper section) and other platforms (lower section) for two real datasets respectively.

Algorithms	R1 ( 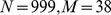 )	R2 ( 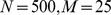 )
MFA (MATLAB)	2.64E3	1.01E3
MCFA (MATLAB)	1.8E3	1.19E3
SSMCL (MATLAB)	43.68	7.18
ULFMM (MATLAB)	0.5	0.38
VBGM (MATLAB)	2.24	1.26
SMART I (MATLAB)	6.4	1.37
SMART II (MATLAB)	**0.47**	**0.37**
DBSCAN (R)	7.44	1.41
MCLUST (R)	165.10	13.44
PFClust (Java)	111.11	35.88

## Methods

### SMART I

Here, we present the implementation of SMART I where OPTOC competitive learning is employed as the splitting and learning algorithm [48], [49], cohesion is employed as merging criterion [54], and MML is employed as clustering selection criterion. The details of how these techniques work together is also presented.

#### OPTOC Competitive Learning

OPTOC competitive learning paradigm was firstly proposed in [48]. In SMART I, OPTOC competitive learning is employed to deal with Task 2. Given each prototype 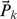, the key technique is that an online learning vector, asymptotic property vector (APV) 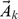 is assigned to guide the learning of this prototype. For simplicity, 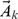 represents the APV for prototype 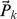 and 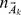 denotes the learning counter (winning counter) of 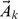. As necessary condition of OPTOC mechanism, 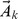 is required to initialize at a random location, which is far from its associated prototype 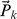 and 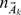 is initially zero. Taking the input pattern 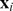 as a neighbour if it satisfies the condition 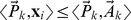, where 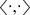 is the inner product operator. To implement the OPTOC paradigm, 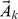 is updated online to construct a dynamic neighbourhood of 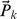. The patterns “outside” of the dynamic neighbourhood will contribute less to the learning of 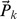 as compared to those “inside” patterns.

In addition to the APV, there is another auxiliary vector, called distant property vector (DPV) 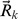, assisting the cluster, which contains more than one prototype, to split. Let 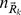 denote the learning counter for 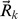, which is initialized to zero. 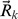 will be updated to a distant location from 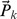. The efficiency of splitting is improved by determining the update schedule of 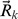 adaptively from the analysis of the feature space. Contrary to the APV 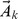, the DPV 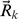 always tries to move away from 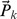. Readers may refer to [48], [49] for the details of updating 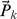, 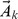 and 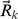.

Original OPTOC claims that the prototype converges if 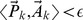. However, 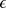 is difficult to determine because it is data related. In our case, we define that the prototype 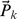 converges if it satisfies 
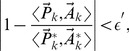
(2)where 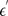 is a positive constant smaller than one. It is worth noting that 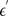 is a relative number and is data-independent. Normally, smaller 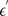 leads to longer learning; while larger 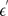 leads to poorer performance. The suggested range of 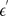 is 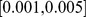. In our experiments, 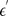 is set to 0.005.

#### Cohesion

In [54], a similarity measure, namely cohesion, was proposed. The cohesion metrics is used for Task 2 in SMART I. It was defined as follows: 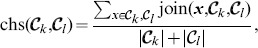
(3)where 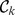 is the cluster with the centroid 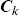, 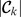 is the size of the cluster of 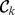. 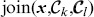 defines the similarity of the two clusters referring to the existence of an object 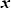, which is defined as 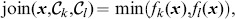
(4)where 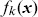 and 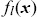 are the probability density function (pdf) of the distributions in clusters 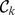 and 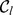. In our case we assume that an object in each cluster follows a multivariate normal distribution.

#### Minimum Message Length

Although there are a lot of model order selection algorithms and validity indices, we choose MML [30], [31], [50] for Task 4 in this work (both SMART I and SMART II) to avoid losing our focus by comparing different selection algorithms. MML is one of the minimum encoding length criteria, like the minimum description length (MDL) [29], [56], and is used as the clustering selection algorithm. The rationale behind minimum encoding length criteria is that if one can build a short code for any given data, it implies that the code is a good model for fitting data. The shortest code length for set 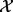 is 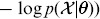, where 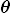 contains the means 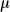 and the covariance matrices 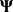. If 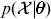 is fully known to both the transmitter and receiver, they can both build the same code and communication can proceed. However, if 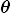 is *a priori* unknown, the transmitter has to start by estimating and transmitting 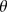. This leads to a two-part message, whose total length is given by 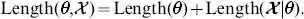
(5)


All minimum encoding length criteria state that the parameter estimate is the one minimizing Length(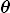, 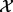). The criterion was derived to the following form [50]

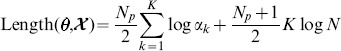


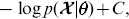
(6)where 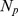 is the number of parameters which is required in each component, 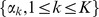 is the mixing probability of the 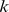-th component with the constraint 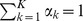, and 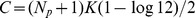 is a constant. Note that the components with zero-probability in 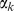 have been eliminated and 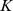 is the number of non-zero-probability components.

#### SMART I Implementation

Here, we integrate these techniques into our SMART framework. The pseudo-code for SMART I is presented in Table 9.

**Table 9:** The pseudo-code for SMART I.

**Task 1:** Initializing SMART with 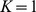
Randomly select 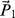 and find the farthest object as 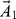 and initialize 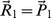 ;
terminate = 0;
**while** !terminate **do**
**Task 2:** Use the OPTOC paradigm for the learning of prototype, and the
splitting of the cluster with largest variance;
**if** the prototype 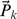 does not converge **then**
Go back to Task 2;
**end if**
**Task 3:** Calculate pairwise cohesions for all converged prototypes (3);
**if** The maximum of cohesions is 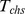 times larger than the median of cohesions
**then**
Merge the pair of cluster with the maximum cohesion;
Go back to Task 3 to continue merging;
**end if**
The stage for recoding candidate clustering.
**if** The number of merges is greater than or equal to 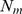 **then**
terminate = 1;
**end if**
**end while**
**Task 4:** Calculate the length for every converged clustering, output the clustering
with the minimum length.

Normally, Task 1 in SMART can be done by any initialization algorithms, either random or deterministic, like the KA algorithm [44]. In SMART I implementation presented here, a simple random initialization is used. The first prototype 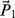 is randomly selected, the APV 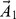 is the farthest object away from 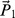, and the DPV 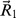 is initialized as 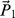. From then on, the SWM process starts. Learning with the OPTOC paradigm drags the prototype to its neighbour, which is “inside” the range of APV, and also drags the APV towards the prototype. Task 2 will not finish until every prototype converges. Since OPTOC is an online learning algorithm, systematic errors may be introduced by the order in which data is fed into the algorithm. Thus, every time OPTOC starts, the order of input data is randomized.

Once the prototypes converge, Task 3 commences. The pairwise cohesions are calculated to measure the distance between the prototype clusters. A criterion is set to guide the merging process, stating that if the maximum of the cohesions is 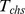 times more than the majority of the cohesions, it reveals that the pair of two prototypes with this maximal cohesion are close enough to merge. The merging process continues until no further merge occurs. A merging counter records the number of merges. After the merging process finishes, the clustering is recorded as the candidate to output. If the merging counter exceeds the maximum number of merges 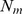, the SWM process is terminated automatically; otherwise, it goes to Task 2 and continues splitting. Once the SWM process finishes, all the candidates are fed into the MML algorithm, which is associated with Task 4, to calculate 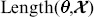. The final clustering results is the one, which minimizes 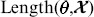.

Note that there are two parameters 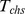 and 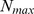 that have to be set in SMART, but they are neutral, i.e., 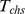 is a relative number rather than absolute one, which is a data-independent value; the reason for setting 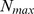 is that normally merging occurs frequently after the natural clustering has been reached. In our experiments, 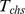 is set to 20 and 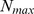 is set to 5. This is the key advantage over those over-clustering-then-merge algorithm, like SSMCL. The critical problem of SSMCL is that if the 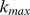 is set too large, some prototypes have possibilities of being trapped in the low density area and difficult to converge.

### SMART II

Here, we present the principal of SMART II, where the finite mixture model (FMM) is employed and the key technique is modified CEM^2^ [50]. Since the FMM and the EM algorithm are very well-known topics, we will not address their details here and readers may refer to [57], [58]. Since the conventional EM algorithm for mixture model has many drawbacks, e.g., it is sensitive to initialization and it is a local greedy method that may be trapped into local minima, the CEM^2^ was proposed in [55] and modified in [50]. The greatest advantage of modified CEM^2^ is that the weaker component may naturally be excluded in the iterative process, which gives the stronger ones a better chance of survival. From the merging point of view, it is a merging process combined with learning.

### CEM^2^ and Its Modification

Clustering dataset 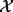, which follows a 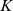-component finite mixture distribution, becomes the discovery of the missing labels 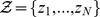 associated with the 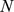 data objects. Unlike conventional EM algorithm, CEM^2^ updates the model parameters 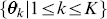 and the probabilities of components 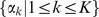 sequentially, rather than simultaneously. In CEM^2^, the estimation is also two-step process, but in each iteration, only one component has the opportunity to update its parameters. For the 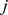-component, it alternates the steps:

- **CEM^2^** ***E-step***: Compute the conditional expectation 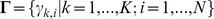 of the missing labels 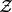 for 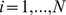 and 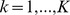, 
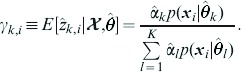
(7)
- **CEM^2^** ***M-step***: Set
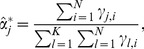
(8)

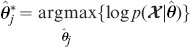
(9)

For 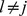, 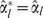 and 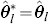.

In [50], the adoption of Dirichlet-type prior for 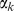
s results a new M-step 
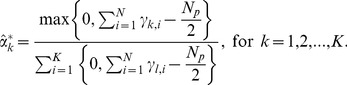
(10)


The corresponding components 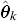
s with 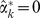 is eliminated and become irrelevant. This component annihilation can be also explained in an estimation theoretic point of view as that the estimates are not accurate unless enough samples are involved. Those estimates without enough samples are dismissed and in turn others have more chances to survive. Modified CEM^2^ can fulfil learning and merging, which are associated with Tasks 2 (only learning part) and 3, respectively, in SMART II.

### SMART II Implementation

Compared with SMART I, SMART II is easier to implement since modified CEM^2^ can do both that are learning and merging. In addition to the learning and merging techniques, there are two configurations different from SMART I. The first is that in SMART II, we initially start with 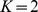 because 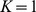 does not need learning, but 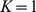 is still included in the candidate list for selection in the output. The second is that the splitting process cannot be done by modified CEM^2^ and has to be specified. Once all components converge and all zero-probability components are discounted, a new component will be injected into the framework. This new component is initialized deterministically by using the farthest object away from the closet component among all the components as the mean and averaged covariance matrix of all components' covariance matrices, as given by 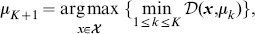
(11)

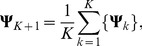
(12)where 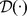 is a distance metric, and then the clustering splits 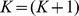. The pseudo-code for SMART II is in Table 10. The stage for recoding the candidate clustering is after all current components converges and all merges finish and before the splitting for new component starts.

**Table 10:** The pseudo-code for SMART II.

**Task 1:** Initializing SMART with 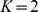
Randomly initialize 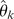 and 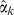 for 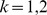 ;
terminate = 0;
**while** !terminate **do**
**Tasks 2 & 3:** Use modified CEM 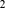 for the learning and merging based on (7) and (10).
**if** the prototype 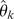 does not converge **then**
Go back to Tasks 2 & 3;
**end if**
The stage for recoding candidate clustering.
**Splitting:** Calculate the parameters for new components (11) and (12);
**if** The number of merges is greater than or equal to 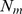
terminate = 1;
**end if**
**end while**
**Task 4:** Calculate the length for every converged clustering, output the clustering with the minimum length.

## Discussion

We have developed a splitting-while-merging (SWM) clustering framework, named splitting-merging awareness tactics (SMART). The framework employs a SWM process and intrinsically integrates many clustering techniques. SMART has the ability to split and merge the clusters automatically during the process. Once the stop criterion is met, the SWM process terminates and the optimal clustering result is selected as final outcome by applying the selection criterion.

Although many recent algorithms have been proposed to achieve automated clustering, e.g. SSCL [48], ULFMM [50], SSMCL [49], PFClust [53], and VBGM [51], [52], there are some issues that limit their practical use. For ULFMM, SSMCL, and VBGM, in spite of the fact that they do not require the exact value of 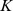, they require the range of 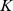, i.e. 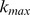, which is also not available sometimes. For PFClust, it needs a good approximation to the distribution of mean intra-cluster similarities, and it requires a large number of randomisation which is time-consuming. The main property of SMART is that it does not require any parameters dependent on respective datasets or *a priori* knowledge about the datasets, particularly, either the number of clusters or the possible range of this number.

### Algorithms

Two SMART algorithms have been implemented with two distinct clustering paradigms: competitive learning for SMART I and learning with finite mixture model for SMART II. Competitive learning is a good candidate technique for on-line learning applications [49]. The selection criterion employs the minimum message length algorithm. It is worth noting that the components in the framework, e.g. the splitting, merging algorithms or the selection criterion, can be replaced by more powerful algorithms in the future, but the whole framework remains unchanged. We summarised the categorization of existing self-splitting-merging algorithms and our two SMART algorithms in Table 1. All existing self-splitting-merging algorithms employ the STM strategy with different clustering paradigms; instead our SMART algorithms employ the SWM strategy. Both algorithms were detailed and tested using demonstration datasets as well as simulated gene expression datasets. We also noted that SMART can be implemented with other clustering paradigms without being restricted in the two techniques presented here. Such flexibility is apparently beneficial to extend SMART to many different applications.

### Effectiveness of the SMART framework

Two demonstration examples illustrated the SWM process and showed the effectiveness of the proposed SMART framework. For different types of clustering techniques, the performance of the SMART algorithms varied. SMART I, for example, did not work well in the D2 dataset, since the CL-based algorithms are spherical. Two models [19], [59], which simulates state-based gene expression data and time-sampled periodic gene expression data respectively, were employed to evaluate the clustering algorithms. In both types of simulated datasets, SMART-II offered remarkably better performance than others. Generally speaking, FMM-based algorithms performed better than CL-based algorithms in these two cases. Furthermore, two real microarray gene expression datasets [7], [65] were studied using SMART. In these experiments, SMART-II also showed superior performance in many metrics. Particularly, SMART II has very small variations in these metrics, which means that it provides consistent results even though it is randomly initialized. Impressively, SMART I has significantly better performance than ULFMM in both real datasets. In the most cases except two demonstration examples, VBGM does not perform well as it is not suitable to directly cluster high dimensional datasets. One major issue of the STM framework, as one of bottom-up algorithms, is that it cannot produce a very accurate clustering result in some circumstances, since it makes clustering decisions based on local patterns without initially taking into account the global distribution. The SWM framework splits and merges the clusters in a top-down fashion to reach a global optimisation.

### Summary

We have proposed a new clustering framework named SMART which possesses three outstanding properties: 1) by integrating many clustering techniques including clustering paradigm, clustering validation, and clustering measure, the proposed SMART framework does not require any parameters dependent on the respective dataset or *a priori* knowledge about the datasets; (2) the implementation of the SMART framework is flexible and extendible to different applications; (3) the SMART algorithms appears to produce more accurate clustering results than counterpart algorithms.

### Future Work

In future work, we will derive new algorithms based on other clustering paradigms, which could be either more robust for general clustering purposes or more appropriate to some particular type of data. Additionally, SMART will be applied in consensus clustering [24], [25], which can achieve consistency among different clustering results of same set of genes in different datasets. Since the critical issue of consensus clustering is the determination of the number of clusters, SMART can overcome this problem and produce different clustering results to many different datasets without specifying any parameters related to respective datasets. Combining these clustering results will reveal consistently co-expressed genes, which have higher possibility to be co-regulated. This can be beneficial in either gene discovery or gene regulatory networks research.
